# Bioinformatics Workflow for Clinical Whole Genome Sequencing at Partners HealthCare Personalized Medicine

**DOI:** 10.3390/jpm6010012

**Published:** 2016-02-27

**Authors:** Ellen A. Tsai, Rimma Shakbatyan, Jason Evans, Peter Rossetti, Chet Graham, Himanshu Sharma, Chiao-Feng Lin, Matthew S. Lebo

**Affiliations:** 1 Personalized Medicine, Partners HealthCare, Cambridge, MA 02139, USA; etsai3@partners.org (E.A.T.); rshakhbatyan@partners.org (R.S.); jason.evans@courtagen.com (J.E.); prossetti@partners.org (P.R.); cgraham@geneinsight.com (C.G.); hsharma@partners.org (H.S.); cflin@partners.org (C.-F.L.); 2 Channing Division of Network Medicine, Brigham and Women’s Hospital, Boston, MA 02115, USA; 3 Department of Medicine, Harvard Medical School, Boston, MA 02138, USA; 4 Department of Pathology, Harvard Medical School, Boston, MA 02138, USA; 5 Department of Pathology, Brigham and Women’s Hospital, Boston, MA 02115, USA

**Keywords:** clinical sequencing, WGS, NGS, next generation sequencing, bioinformatics, validation, precision medicine

## Abstract

Effective implementation of precision medicine will be enhanced by a thorough understanding of each patient’s genetic composition to better treat his or her presenting symptoms or mitigate the onset of disease. This ideally includes the sequence information of a complete genome for each individual. At Partners HealthCare Personalized Medicine, we have developed a clinical process for whole genome sequencing (WGS) with application in both healthy individuals and those with disease. In this manuscript, we will describe our bioinformatics strategy to efficiently process and deliver genomic data to geneticists for clinical interpretation. We describe the handling of data from FASTQ to the final variant list for clinical review for the final report. We will also discuss our methodology for validating this workflow and the cost implications of running WGS.

## 1. Introduction

Precision medicine is becoming an increasing focus in medical research [1]. To achieve the resolution necessary to personalize clinical care, greater attention has been drawn towards higher resolution of the patient genome. Next generation sequencing (NGS) provided a cost-effective method for targeted sequencing of known disease genes at base pair resolution [2]. Moreover, the advent of exome sequencing enabled rapid discovery of genes causing Mendelian disorders. While gene panels and exome sequencing have proved fast and cost-effective for delivering genomic results back to the patient, these technologies are limited by our current knowledge of the exome, which changes over time. Additionally, the use of targeted capture may introduce biases to the data, including PCR duplicates, depth of coverage disparities, and failures at difficult to amplify target regions [3].

Practical considerations such as sequencing costs, data processing and maintenance, and data analysis complexities are important considerations when a laboratory is considering a new NGS program. These issues are amplified in whole genome sequencing (WGS) due to the volume of the data and have long been barriers to entry for clinical laboratories looking to adopt WGS. Despite the ability of WGS to interrogate the entirety of the genome, clinical interpretation still often focuses on only 3% of the genome (*i.e.*, exome data, pharmacogenomics risk variants, and single nucleotide variants associated with complex disease risk) [4,5,6,7]. Therefore, WGS services may be overlooked for clinical applications as they trend towards increased costs and longer turnaround times due to a heavier computational load, increased number of variants for analysis, and larger data archives. However, the steadily decreasing cost of sequencing and storage now allow laboratories to consider genome sequencing. WGS, and more specifically PCR-free WGS, also decreases the need to re-sequence each time the coding sequences of targeted regions change, novel genes are discovered, or a new reference genome is released. The balance between cost, turnaround time, accuracy, and completeness has to be addressed when launching a WGS program. Here, we describe the workflow we adopted and the challenges we met supporting the bioinformatics of WGS in a clinical setting.

## 2. Results

### 2.1. Bioinformatics Validation

Our pipeline performs robustly as different entry points and different runs of the same sample returned exactly the same variants. Using HapMap sample NA12878 and previous Sanger confirmed regions, we identified thresholds for variants of Quality by Depth (QD) ≥ 4 and Fisher Strand Bias (FS) ≤ 30 as providing the optimal balance of sensitivity and specificity. This sample has been well characterized by our laboratory and also by other groups [8,9]. Of 425 total confirmed variants, all 425 variants were detected by genome sequencing for a sensitivity of 100% (95% CI: 99.1%–100% for SNVs and 79.6%–100% for indels; Table 1a). In addition, four likely reference sequence errors, positions where only the alternative allele has ever been identified, were correctly genotyped by genome sequencing as homozygous for the alternative allele, three of which had incorrect genotype calls with previous orthogonal assays. In addition to these true positive variants, calls were also made for 21 false positive (FP) variants, including 20 substitutions and one indel (Table 1b). After filtration of variants based on optimal QD and FS thresholds discussed, only one FP remained.

Our genome-wide comparison of the variants detected through this pipeline compared to those detected at a similar coverage through the 1000 Genomes Project for NA12878 confirmed similar results (Table 1c). Out of 2.7 million SNVs and 285,000 indels detected through our pipeline, we found that 98.8% of these SNVs were also called in the 1000 Genomes dataset. In addition, 97.6% of the total variants called were concordant for genotype. Annotation and variant filtration were also performed on NA12878. Greater than 50 variants were randomly selected for manual inspection to ensure that they were properly annotated and filtered.

### 2.2. Known Regions of Poor Coverage 

All of the genomes delivered to us from Illumina’s CLIA-laboratory have at least 30X coverage, with an average coverage of 43X. However, this coverage varies across the coding sequences of the genome and affects both clinically relevant and clinically unknown regions of the genome. A gene-level list of the percentage of callable bases is provided in Supplementary Table S1. In total, of the 1381 genes with at least five asserted Pathogenic or Likely pathogenic variants by clinical laboratories in ClinVar, 94 had <90% coverage across their coding region. The 20 genes with the poorest coverage metrics included many with high prevalence and clinical relevance (Table 2). In our clinical process, for indication gene-list driven analyses, we report back the coverage of analyzed genes, highlighting those with coverage issues. This provides a useful guide to determining potential false negative findings, including those due to regions that are difficult to sequence with NGS technologies.

### 2.3. Cost Analysis and Scalability

The majority of our bioinformatics costs of WGS lies in data storage and not in computational processing. As part of our clinical workflow, the FASTQ, BAM, and VCF files are periodically archived on a replicated, secondary storage site. We also store MD5 checksums to ensure data consistency during transfer and storage. These disks are primarily used to store data, and any access to these files must be done after they are copied back onto a faster storage location (e.g., primary storage). Based on the calculations of CPU time used and the lifespan of our cluster as well as the hourly costs of a bioinformatics analyst, we estimated that it costs $77 for the computational time and $128 for the hands-on time necessary to process a genome. However, approximately 300 GB per genome of storage is required to keep files containing unaligned and aligned reads as well as the final variant calls (respective FASTQ, BAM, and VCF files). In Table 3, we estimate the annual cost of storage per genome to be around $40–$55, depending on whether the genome is stored on a primary storage device or a secondary (or archival) disk; the bioinformatics process and storage of a genome for one year for our laboratory is approximately $245. Disk storage of sequencing data incurs significant cost in addition to the sequencing and interpretive components, and depending upon laboratory needs and policies, the availability of the data on either active or deep storage may differ. This pipeline is built on a high performance computing (HPC) cluster, so the ability to process many genomes at a given time is heavily dependent on the size of the cluster. With our current infrastructure, we do not run more than 10 genomes simultaneously. The pipeline is scalable with respect to the cluster size, but we have not explored scaling this process to simultaneously handle hundreds or thousands of genomes.

## 3. Discussion

The validated bioinformatics workflow we described above has been used to process hundreds of genomes. We have been updating its functionality and re-validating the components of this process multiple times since initially implementing the pipeline. The modularity of our process allows us to repeat only the annotation, upload, and filtration processes when new or updated annotation resources are available. Similarly, filtrations can be easily re-queried from the persistent variant data in the Oracle database.

We have also compared the results of our NA12878 with the Genome in a Bottle Consortium (GIAB) dataset driven by the National Institute of Standards and Technology (NIST) [9]. A similar tool known as the Genetic Testing Reference Materials Coordination Program (GeT-RM) allows users to query across different alignments of the same dataset to see if the variant of interest found by your pipeline also is found by other pipelines. This dataset includes variant data generated by our laboratory. Both of these resources are tremendously valuable in validating clinical genome sequencing.

The limitation of genome sequencing is an important consideration. Although it is often times termed “whole genome,” it has many regions that cannot be accurately determined by current technologies. These regions are inaccessible to current, standard clinical WGS analysis and include regions of high homology for which reads cannot be uniquely mapped, regions where the reference genome contains errors, regions with multiple reference haplotypes, and tandem repeats that extend beyond the sequenced read length. These regions can overlap with clinically relevant areas and can potentially lead to false negative results if not dealt with carefully. In Table 2, we have indicated a subset of these that consistently have the lowest coverage in our WGS assay. Current techniques for managing these regions include repeat-primed PCR for repeat expansion disorders, long-range PCR for genes with homology issues, and allele-based barcoding techniques to anchor reads to unique neighboring regions. Aligning to the GRCh38 reference genome may help for regions with multiple reference alleles, but it is still unclear how those regions will be implemented in a clinical workflow. Eventually the use of longer read technologies, and possibly *de novo* assembly, will enable more accurate aligning and detection of variants in these difficult regions.

As genome sequencing becomes more routine, additional enhancements will continuously be made to the process. In our laboratory, these enhancements focus on a user interface for variant filtration to enable live filtering for our clinical staff, identification of copy number and structural variation from the WGS data, and upgrading the pipeline to align to the GRCh38 reference genome. This work requires a team of bioinformaticians with a broad skillset that can interact directly with both the clinical staff and the information technology teams. Finding this dynamic can prove to be one of the most challenging aspects of creating a clinical genome sequencing program.

## 4. Methods and Materials

### 4.1. Bioinformatics Pipeline

The bioinformatics pipeline processes data from the hard drives delivered by Illumina Clinical Services Laboratory (San Diego, CA, USA) to the variant report files generated for clinical interpretation in a fairly automated fashion. As these hard drives are received, they are accessioned in the laboratory and uploaded to our high performance computing (HPC) environment. The BAM files delivered on the hard drives contain reads aligned by the pipeline developed at Illumina using CASAVA (or ISAAC in recent data deliveries). These files contained both aligned and unaligned reads, so the conversion from BAM back to FASTQ format to decouple mapping information from the read sequence does not incur loss of sequencing information.

**Figure 1:**
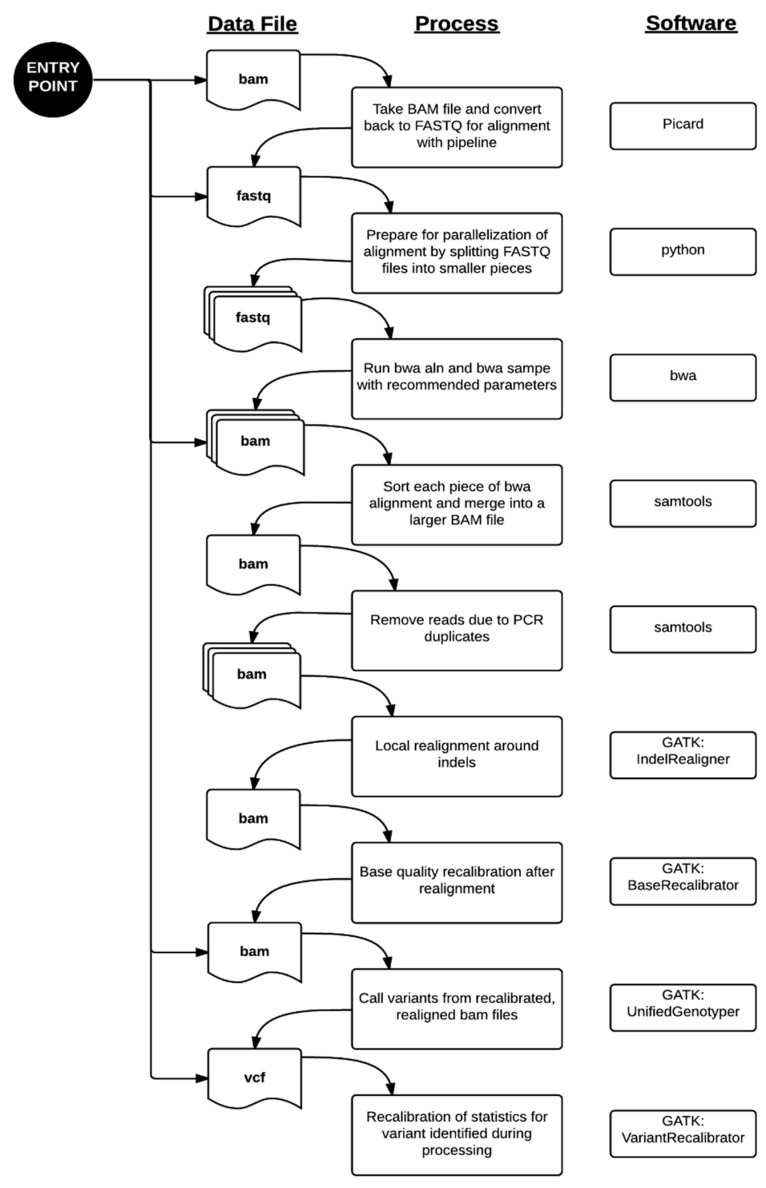
WGS Alignment and Variant Calling Pipeline. There are multiple entry points to our pipeline where it can be re-triggered due to system failures or outside datasets. Standard processing of genome data from the Illumina Clinical Services Laboratory starts at the top entry point. From there, FASTQ sequences are aligned to the reference hg19 genome using the burrows-wheeler aligner (bwa). Since the alignment is computationally intensive, we divided the sequence files into smaller files. The alignments, known as “raw” BAM files in our pipeline, are processed through a series of steps prior to variant calling. The “final” BAM file is the resulting file after removing PCR artifacts, local indel realignment, and base quality recalibration. This is used as the input file to the variant caller portion of the pipeline. Variant calling happens in two phases, where the variants are identified and then their quality scores are recalibrated in the final VCF file.

Data processing from the FASTQ file to the VCF file was performed primarily using the parameters recommended by each respective software package [10,11,12,13]. Paired-end alignment of the sequencing reads to the hg19 reference genome was performed using bwa 0.6.1-r104. The aligned reads were sorted and PCR duplicates removed using samtools 0.1.18. Local indel realignment, base quality recalibration, variant calling by UnifiedGenotyper, and variant recalibration were performed using Genome Analysis ToolKit (GATK) 2.2.5 and the recommendations in the Best Practices Workflow by the GATK development team at the Broad Institute (Cambridge, MA, USA). The entire workflow is summarized in Figure 1. For more efficient computing, our validated process includes parallelization by dividing files into smaller pieces for even distribution across the cluster. This method was described in our application of this pipeline to the MedSeq Project, a randomized clinical trial assessing the impact of genome sequencing in clinical practice [14] and to the discovery of a putative locus causing a novel recessive syndrome presenting with skeletal malformation and malignant lymphoproliferative disease [15]. Similar pipelines have been used successfully in other clinical labs using NGS previously [4,6,16,17,18].

Successive variant annotation is accomplished through a set of scripts that independently annotate the dataset with a wide collection of information, including: (1) transcript and gene annotations from Alamut (Interactive Biosoftware, Rouen, France); (2) gene annotations from variant effect predictor (VEP); (3) variant annotations from 1000 Genomes Project, ClinVar, and Exome Sequencing Project (ESP); and (4) clinical interpretation from our laboratory maintained in GeneInsight (Cambridge, MA, USA) [19,20,21,22]. As a clinical laboratory, we are focused on genomic regions that are known or likely to be associated with disease. Thus, we have limited the annotation of variants called on the genome to only those that lie in our target regions. The target region contains the RefSeq coding sequence for all genes mapped to hg19, pharmacogenomic variants, and association or risk variants taken from the NHGRI GWAS catalog [23]. These regions are buffered by 50 base pairs (bp). The VCF file is limited to only this target region prior to variant annotation. Additionally, during filtration and coverage analysis, we focus in on the regions most likely to contain clinically reportable disease causing variants. Thus, we created clinical region of interest (ROI) files containing all coding regions of all exons for each gene ±15 bp or ±2 bp for filtration and coverage, respectively.

**Figure 2:**
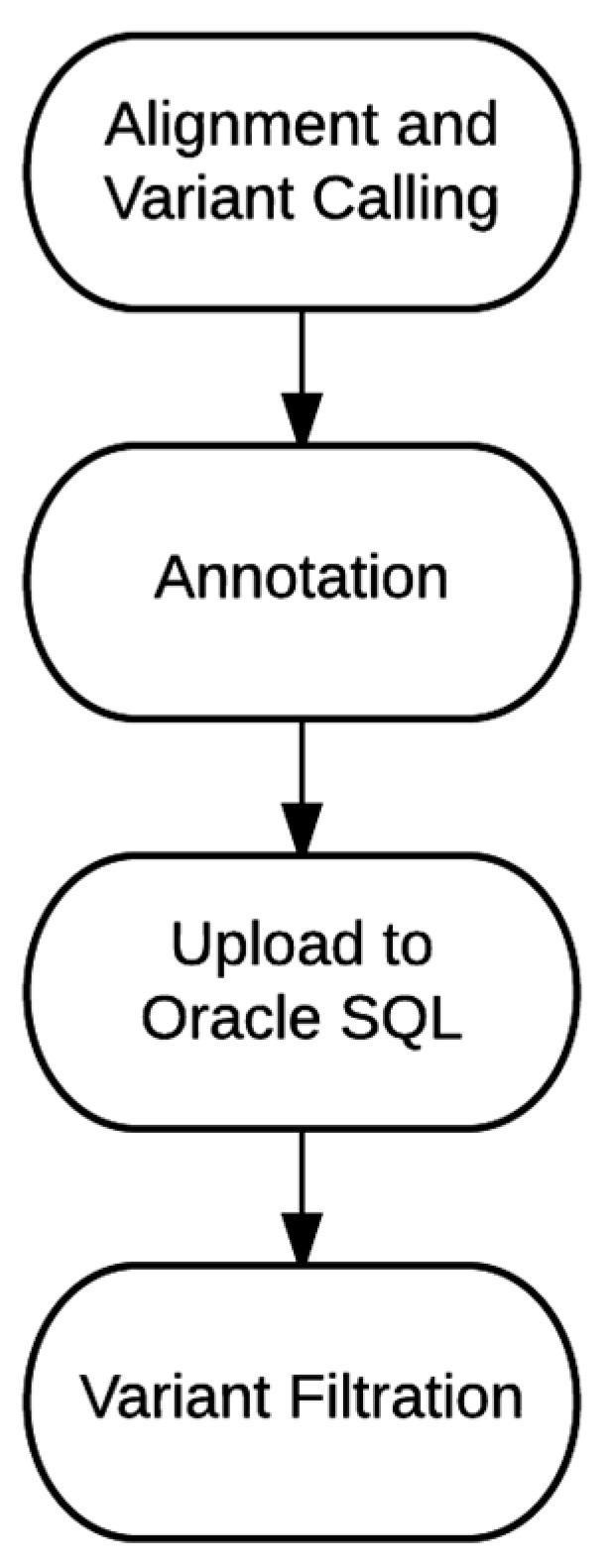
Bioinformatics Workflow. Our process is divided into four major phases. During this process, there are three trigger points that require manual hands-on time: (1) Alignment and Variant Calling; (2) Annotation and Upload to Oracle SQL; and (3) Variant Filtration. Segmenting these processes offer the ability to check the data integrity throughout this process and the flexibility of utilizing parts of these scripts for processing a non-standard clinical or research sample.

The sample data and variant annotations are uploaded to an Oracle SQL database. The variant filtration process was designed to query this database and return the variants of interest in an excel spreadsheet for careful clinical review. Variant filtration has been an evolving process, and filtration specifications vary depending on the prevalence, penetrance, and predicted mode of inheritance of the genetic disorder. Table 4 lists some of the common filtrations used to generate the final report. Often, the final variant report is generated using Boolean logic to combine more than one of these filters. The overview of the entire bioinformatics process, from alignment and variant calling to final report generation is outlined in Figure 2.

### 4.2. Bioinformatics Validation

The WGS dataset of NA12878 retrieved from Coriell Cell Repositories (Camden, NJ, USA) and sequenced at Illumina was used to validate the efficacy of this pipeline. The pipeline was run twice on the exact same dataset to test the robustness of the pipeline, proving it can consistently deliver the same results for a given dataset. For this test, the pipeline was triggered from two different upstream entry points, Illumina-aligned BAM files and FASTQ files, and the final variant output files of this test were compared. Another source of validation comes from understanding the sensitivity and specificity of the variant calling pipeline. These metrics were estimated using different standard datasets that have assessed the genome level variation on NA12878. The variant calls were first compared to internally generated sequencing data. NA12878 has been the validation standard of many laboratories, and we have sequenced about 700 kb of sequence across 195 genes from our cardiomyopathy, hearing loss, respiratory, Noonan syndrome, and Marfan syndrome tests using a combination of orthogonal technologies including Sanger sequencing, array-based sequencing, and targeted next-generation sequencing [2,24,25]. Consequently, we estimated sensitivity and specificity of our WGS workflow using only this specific region of interest. As recommended for earlier versions of GATK, we varied the QD and FS filters for variants called to find the optimal threshold. We additionally checked the concordance rate of our genome-level data against the variants in NA12878 reported by the 1000 Genomes Project dataset of high coverage WGS (>30× average coverage) [19]. The variants were assessed by their presence in each VCF file (variant concordance) as well as the concordance of the genotype called for each variant (genotype concordance).

### 4.3. Characterization of Poorly-Covered Regions

We selected 15 genomes sequenced at Illumina Clinical Services Laboratory and processed through our bioinformatics pipeline in FY2014 to generate a coverage list for the coding sequence of RefSeq genes plus 2 bp of buffer to encapsulate splice sites. We customized GATK 2.2.5 to run CallableLoci to determine the callability of every coding base pair, where “callable” is defined as a base having at least 8X of coverage from quality reads and less than 10% of the total reads in that region have poor mapping quality. The average of the callable base pairs for each gene across 15 genomes was calculated for this coverage list. Our pipeline did not perform variant calling on chrMT, so mitochondrial genes were excluded from this list.

Next, we wanted to assess the clinical relevance of the poorly covered regions identified in the coverage list. To do so, we selected the ClinVar dataset of reported variants, a public archive of variants and their clinical significance as contributed by the medical genetics community [26]. We selected “Pathogenic” and “Likely pathogenic” variants interpreted by the clinical significance element in the XML release of the ClinVar dataset (August 2015 release). ClinVar has several categorizations of variants, and we define a unique variant as one having a unique MeasureSet ID. To identify clinically relevant genes that are currently assayed in clinical sequencing laboratories, we focused on variants submitted by laboratories and working groups. To do this, we removed variant classifications from large, research-grade databases, including OMIM. MeasureSet IDs with submissions of conflicting clinical significance, such as being reported as pathogenic by one laboratory but benign in a different laboratory, were also excluded. However, an assertion of pathogenic in one laboratory but uncertain significance in another laboratory would be consistent and therefore not counted as a conflicted variant.

### 4.4. Cost Analysis and Scalability

WGS is more computationally intensive and demanding of storage than other current sequencing solutions. It incurs additional costs as clinical sequencing requires the data to be preserved for a set amount of time. The computational costs were estimated by the price of purchasing the compute nodes adjusted for the amount of CPU-hours necessary for a genome run and the lifespan of the hardware, which is guaranteed for five years at Partners HealthCare. The hands-on time was estimated by the price we charge per hour for bioinformatics analysis multiplied by the time necessary to successfully trigger the pipeline, deliver the variants to the geneticists, and archive the data. We also calculated the cost of storage based on current internal prices offered. The cluster resources used to run the genome sequencing pipeline is shared across many bioinformatics processes. At present, we have 10 compute nodes, each with 128 GB RAM and 16-core Intel Xeon 2.60 GHz processors running CentOS 6.5 and IBM LSF job scheduler.

## 5. Conclusions

We have described the implementation of clinical WGS in our laboratory at Partners HealthCare Personalized Medicine. Our implementation includes specialized scripts to perform: (1) alignment and variant calling; (2) variant annotation; (3) importing data to an SQL database; and (4) variant filtration. Each of these components has been clinically validated and can be updated independently of each other. WGS has been shown to reliably identify genetic variants, but the main challenge remains in clinical interpretation of those variants. This is particularly true for the vast majority of non-protein-coding variants, which are largely omitted in whole exome sequencing. With a larger public knowledgebase on non-coding regions, such as ENCODE data and GWAS signals, we can begin to create filtrations that include larger portions of the non-coding regions as they start to have utility in the clinical setting, ultimately enabling the full utility of complete genome sequencing.

## Figures and Tables

**Table:** (**a**) Specificity

Variant Type	FP (before Thresholds)	FP (after Thresholds)
SNVs	20	1
Indels	1	0

**Table:** (**b**) Sensitivity

Variant Type	#	FN	Sensitivity	95% Cl
SNVs	410	0	100%	99.1%–100%
Indels	15	0	100%	79.6%–100%

**Table:** (**c**) Concordance with 1000 Genomes data

Variant Type	1K Genomes Variants	Present in NGS Calls	% Present in NGS Calls	Present in NGS Calls with Matched Genotypes	% Present in NGS Calls with Matched Genotypes
SNVs	2,762,933	2,735,592	99.01%	2,730,826	98.84%
Indels	3,27,474	299,300	91.39%	285,401	87.15%
Total	3,090,407	3,034,892	98.20%	3,016,227	97.60%

**Table 2:** Top 20 Poorly Covered Genes with Clinical Relevance. Clinical relevance is defined as having at least five Pathogenic or Likely pathogenic variants in ClinVar reported in the gene by submitting laboratories or working groups.

Gene	# Clinically Significant Variants	% Callable	Disease	Disease Prevalence
STRC	8	20	Sensorineural hearing loss	Common
ADAMTSL2	5	32	Geleophysic dysplasia	Rare
CYP21A2	13	44	Congenital adrenal hyperplasia	Common
ARX	19	45	X-linked infantile spasm syndrome	Rare
MECP2	250	53	Rett syndrome	Common
GJB1	16	53	Charcot-Marie-Tooth disease	Common
ABCD1	33	57	X-linked adrenoleukodystrophy	Moderate
EMD	11	57	Emery-Dreifuss muscular dystrophy	Moderate
G6PD	16	58	Glucose-6-phosphate dehydrogenase deficiency	Common
GATA1	12	60	Dyserythropoietic anemia and thrombocytopenia	Rare
AVPR2	15	62	Nephrogenic diabetes insipidus	Rare
EDA	37	63	Hypohidrotic ectodermal dysplasia	Moderate
SLC16A2	11	63	Allan-Herndon-Dudley syndrome	Rare
FLNA	42	64	Otopalatodigital syndrome	Rare
EBP	24	64	X-linked chondrodysplasia punctata	Rare
RPGR	17	64	Retinitis pigmentosa	Common
TAZ	17	64	Barth syndrome	Rare
IDS	16	64	Hunter syndrome	Moderate
FGD1	8	64	Aarskog-Scott syndrome	Rare
GPR143	6	65	Ocular albinism	Moderate

**Table 3:** Cost Analysis for Storage of WGS data. Primary storage assumes unreplicated, active storage with high input/output (I/O) capacity. Secondary storage assumes replicated, deep storage with low I/O capacity. The cost of processing a genome and data retention on the primary and secondary storage for one year is ~$245.

Storage Type	Genome/Month ($)	Genome/Year ($)	Genome/5 Years ($)
Primary	4.42	53.04	265.20
Secondary	3.48	41.76	208.80
Total	7.90	94.80	474.00

**Table 4:** Example Filtration Methods. These filters are applied using Boolean logic to produce the final list of filtered variants in each individual.

Filter Name	Parameter	Description
Frequency	X (e.g., 0.01 or 0.05)	Keep variants that have frequencies in ESP or 1000 Genomes ≤ X
Loss-of-Function		Keep variants that may implicate loss of gene function, including those annotated with the following Sequence Ontology keywords: frameshift_variant, stop_gained, stop_lost, splice_acceptor_variant, initiator_codon_variant, splice_donor_variant.
Gene List	Gene list (in HGNC nomenclature)	Gene filtration is based on selecting variants that are within particular genes. We check if a variant is annotated with a gene symbol of interest within a clinical region of interest
Reported Pathogenic		Select variants that are classified as Pathogenic or Likely pathogenic in variant databases, including ClinVar
GeneInsight		Select variants that are classified as Pathogenic or Likely pathogenic in our internal GeneInsight database
Compound Heterozygous		Select LOF and missense variants if there are at least two alterations in the gene that may impact function of both alleles

## Supplementary Materials

Supplementary materials can be accessed at: http://www.mdpi.com/2075-4426/6/1/12/s1.
